# The Effect of Selenium Administration on Restless Leg Syndrome Treatment

**Published:** 2012-01-01

**Authors:** A G Rahimdel, P Ayatollahi, A Zeinali, N Mehrabanian, A Mellat-Ardekani

**Affiliations:** 1 Department of Neurology, Shahid Sadooghi Hospital, Yazd University of Medical Science, Yazd, Iran

**Keywords:** Restless leg syndrome, Selenium, Treatment

## Abstract

**Background:**

Restless leg syndrome (RLS) is defined as an uncomfortable feeling in the limbs which is prominently sensed in legs. Dopamine system involvement is considered as the base of RLS's etiology. Because of safety, anti-oxidant and dopaminergic promoting action of selenium, this study aims to investigate the effect of selenium on restless leg syndrome treatment.

**Methods:**

Sixty patients with primary RLS were enrolled in this clinical trial (Irct2011103015943n1). It was based on 3 periods of drug prescription with one month wash out period. As placebo, 50 and 200 μg of selenium were administered in each separated month. The diagnosis was based on criteria published by IRLSG (International RLS Study Group). The questionnaire included 10 questions while each question’s rating was between 0 and 4. Points between 1 and 10 were considered mild, 11 to 20 as moderate, 21 to 30 as severe and 31 to 40 as very severe. After end of each month of drug consumption, questionnaires were completed and each subject was asked to report the severity of disease and side effects of the drugs. At least 10 declines in scale were considered as appropriate responses.

**Results:**

Improvement (decline IRLS score >10) was significantly higher in selenium (50 and 200 μg) than placebo group.

**Conclusion:**

Selenium prescription in daily recommended dose of 50 μg instead of a dopamine agonist would be an alternative treatment in improvement of RLS symptoms.

## Introduction

Restless leg syndrome (RLS) is defined as an uncomfortable feeling in the limbs which is prominently sensed in legs and rarely in arms.[1] This disturbing feeling leads to restless and forces patient to move the limbs which is pathognomonic manifestation of RLS.

This movement leads to complete but temporary relief of symptoms. These symptoms occur only in rest and are intensified in evening or night time.[1] The prevalence of disease was shown to be 5-10%.[1][2][3] and the prevalence increased by aging.[3] In addition to sensory symptoms, motor symptoms such as periodic limb movement syndrome, myoclonic Jerking exist during the patient's sleep.[1][2] Since these symptoms may occur during sleep, most of patients have some difficulty in start of sleeping or during sleep which may lead to fatigue and excessive daily sleepiness. Thus RLS is one of the most important causes of insomnia and sleepiness during the day.[4] The etiology of RLS is not precisely defined yet but it may be primary (idiopathic) or secondary. Primary cases are commonly genetical and are presented as autosomal dominant patterns.[5][6][7][8] Secondary types are associated with diseases such as iron deficiency anemia, folic acid deficiency, renal failure and physiological conditions such as pregnancy.[1][9] Also some case reports showed an association between RLS and diabetes melitus, rhematoid arthritis and amyloidosis.[1][9]

RLS may be induced secondary to drug consumption such as lithium, three cyclic antidepressants, dopamine blockers like metoclopramide and phenothiazines.[1][2][3] Some facts such as response to dopaminergic drugs, drug induced RLS like anti-dopaminergics, hypoactivity of dopamine system, decreased connection to dopamine receptors in basal ganglia[4][10][11] implicate this hypothesis that dopamine system involvement may be the main cause. The diagnosis is clinical and the most important role in diagnosis is the history of uncomfortable feeling in limbs which occurs only at rest and is aggravated in evening and night time and is relieved by limb movement.[1][11] Diagnostic criteria published by IRLSG (International RLS Study Group) were shown in Table 1.[12] Some laboratory studies are necessary for diagnosis completion including renal function test, cell blood count, serum iron, ferritin and folic acid levels, EMG-NCV and polysomnography for periodic limb movement syndrome(PLMS).[1][4][11] In recent years, questionnaire for diagnosis and severity determination of RLS has been established by Benoy and Kohnen which is a gold standard for the diagnosis and severity determination. The questionnaire includes 10 questions while each question is rated between 0 and 4. Points between 1 and 10 are considered mild, 11 to 20 as moderate, 21 to 30 as severe and 31 to 40 cas very severe.[4][9][13][14] Differential diagnosis are peripheral neuron diseases such as painful peripheral neuropathy, vascular diseases such as peripheral vascular diseases, varicose and erythromelalgia, movement disorders such as akathisia, extra pyramidal tremor, etc.[1][4][11]RLS treatment is based on two approaches including pharmacologic and non-pharmacologic. Nonpharmacologic approaches include treatment of the underlying causes like iron supplementary consumption in iron deficient patients, kidney transplantation in uremic patients, drug induced RLS withdrawal, prevention of stimulating subject consumptions such nicotine, alcohol and caffeine. Also sleep hygiene has an important role in reduction of the disturbances.[1][4][9]Pharmacologic approaches include dopaminergic drugs such as levodopa,[9][15] pramipexole,[9] ropinrinol[2][9][15] and pergolide,[2][15] benzodiazepines,[15] opioids such as propoxyphen subsulicylate,[2] antiepileptics such as gabapentin,[2] iron and folic acid.[15]

**Table 1:** Diagnostic criteria for RLS

***Essential criteria***	
1.	An urge to move the legs usually accompanied by uncomfortable and unpleasant sensation in the legs.
2.	The urge to move or unpleasant sensations to be begun or worsen during periods of rest or inactivity such as lying or sitting.
3.	The urge to move or unpleasant sensations to be partially or totally relieved by movement such as walking or stretching at least as long as the activity continues.
4.	The urge to move or unpleasant sensation to be worsen in the evening or night than during the day or only to be occurred in the evening or night.
Supportive features	
1.	Positive family history
2.	Positive response to dopaminergic therapy
3.	Periodic limbs movements
Associated features	
1.	Natural clinical course
2.	Sleep disturbance
3.	Medical evaluation/physical examination.

Selenium was discovered by Brezilus in 1814.[16] In 1957, Foltz and Schwarz implicated that selenium is a rare essential element existing in dietary regimen and has a preventive role for the diseases.[17] Body selenium concentration is related to its soil concentration.[18][19] This concentration in some countries like United States of America is high and is low in China.[20][21][22] In 1973, Rotrack and colleagues demonstrated the anti-oxidative role of selenium.[15] Role of selenium as anti-oxidant and anti-inflammatory in reverse striatal dopamine depletion[23] and decrease in thyrosine hydroxylase activity were shown.[24][25] Selenium is administered by two routes in selenium deficient patients including enteral and parenteral. Also it is prescribed as supplementary diet in pregnant or breast-feeding mothers.[26] In advised doses, no side effects were reported. Even no intoxicated cases with doses up to 400 mg for prostate cancer prevention were shown.[27][28][29][30] Because of its safety, and anti-oxidative and dopaminergic promoting actions and dopaminergic agonist side effects, we were encouraged to establish this study to determine the effect of selenium in RLS treatment.

## Materials and Methods

In our study, which began in June 2010 and lasted until November 2010, initially 68 patients were enrolled with diagnosis of primary RLS based on IRLSSG. The trial was an experimental study and was registered in IRCT (Code: Irct2011103015943n1). All patients passed the full exams by two neurologists. None of examiners had any knowledge about the colleague's diagnosis. Patients diagnosed as primary RLS by two neurologists were enrolled. The inclusion criteria were (i) Severity of symptoms at least to be moderate (point >10 based on IRLS) and (ii) Patients to be in primary RLS category. All patients underwent routine laboratory exams too. After confirmation, primary cases were enrolled. Eight patients were omitted during the study because of low compliance and insufficient data feedback and finally 60 patients passed the clinical trial successfully. All patients signed an informed consent and were informed for steps of clinical trial and the probable side effect of the drug. The trial was based on 3 periods of drug prescription (every period of the last one month) with one month wash out period. Only the designer of study was aware of the order of drug administration and the others (neurologists, drug administrators and patients) were blind to the pattern and order of drug administration. Table 2 shows the steps of trial.

**Table 2:** Steps of trial

**State**	**Placebo**	**Washout**	**50 μg selenium**	**Washout**	**200 μg selenium**
Duration	One month	One month	One month	One month	One month

Placebo, 50 and 200 mg selenium were prescribed as capsules in equal shape and form. Patients and investigators did not know about the materials inside the capsules. The capsules were delivered to patients in three distinctive same packages with information insides, including start and end of the data. During the trial, all patients were questioned regularly about the consumption and dose effects of the medication and were encouraged to use the drugs precisely. After the end of each month of drug consumption, questionnaires were completed and asked them to describe the severity of the disease and any side effects of the drug. At least 10 declines in scale were considered as an appropriate response. All statistical analyses were performed using SPSS software (version 11.5, Chicago, IL, USA).The results were shown as mean±SD. In every period, before-after scores of males and females were analyzed separately by using nonparametric Wilcoxon test. Comparison of the effect of the drug in every period between males and females was assessed by Mann-Whitney test. All before-after scores in the three groups were compared together by Freedman test. P-value of < 0.05 was considered as statistically significant.

## Results

Sixty patients (34 females, mean age: 54±11.4 and 26 males, mean age: 49±12.3) were enrolled. Table 3 shows the rate of placebo and selenium efficacy. Rate of improvement (decline IRLS score>10) was signifi­cantly higher in selenium than placebo group. Im­provement rate between 50 and 200 mg of selenium consumption was not significantly different.

**Table 3:** Score decline comparison between placebo and 50 and 200 µg of selenium.

**Score decline**	**Placebo No. (%)**	**50 µg selenium No. (%)**	**200 µg selenium No. (%)**
10-15	11 (18.3)	27 (45)	30 (50)
15-20	0	4 (7)	6 (10)
Total	11 (18.3)	31 (52)	36 (60)

Patients score before and after treatment with pla­cebo, 50 and 200 μg of selenium were shown in Table 4. There were no significant differences between males and females in response to placebo, 50 and 200 μg of selenium (p values respectively=0.489, 0.473 and 0.446). Decline in score after treatment by 50 and 200 μg of selenium was significantly higher than pla­cebo group (Both p values were less than 0.001). Also difference between 50 and 200 μg of selenium was significant (p value=0.007).

**Table 4:** Score comparison before and after treatment.

**Score mean(SD)^a^**	**Placebo**			**50 µg**			**200 µg**		
	**Before**	**After**	***P* value**	**Before**	**After**	***P* value**	**Before**	**After**	***P* value**
Male	27.73 (6.91)	21.96 (5.32)	<0.001	26.32 (5.34)	13.50 (4.24)	<0.001	25.65 (5.09)	11.63 (4.57)	<0.001
Female	29.21 (6.34)	22.87 (5.71)	<0.001	27.84 (4.86)	14.95 (4.52)	<0.001	27.23 (4.91)	13.19 (3.82)	<0.001
Total	28.57 (5.82)	22.48 (4.32)	<0.001	27.03 (4.67)	14.17 (4.02)	<0.001	25.89 (5.44)	11.86 (4.62)	<0.001

^a^ SD: Standard Deviation

## Discussion

In our study, females were more afflicted by primary RLS than males (34 vs. 26).This may be due to sexual hormone effects, dietary regimen, psychologic and sleep problems in this gender. Also silent coincidence disease in females may be more than males which we could not find them precisely.

Table 5 shows the comparison between our study and other results, using RLS score for RLS responses to treatment. According to Table 5, in most studies, the rate of score decline by placebo ranged between score of 5-8. The role of placebo was investigated in declining score of RLS.[32] Increasing the opioid and dopaminergic system of brain may play an important role.[32] Maximal decrement in score was correlated to dopaminergic agonists (17.2 by trans-dermal roting-tone), but the interesting fact was that despite the more declines in scores by dopminergics, loss of side effects by selenium consumption and safety use of selenium and score decline to 12.86, less compliance and more cost of dopaminergics, selenium may be more and well tolerated. Several studies implicate the role of selenium in reconstruction of the dopaminergic system.[23][24][25] Score declines by 50 and 200 µg of selenium were 12.86 and 14.03 respectively and this difference was statistically significant (p=0.007) but practically this difference was not cost effective. So we preferred to choose 50 µg of selenium for RLS treatment.

**Table 5:** Comparison of IRLS score decline between studies

**Study**	**Country**	**Year**	**IRLS score decline**		**Price in Iran (Consumption dose per day)**
			**Placebo**	**Drug**	
Rahimdel	Iran	2010	6.09	12.86 (Selenium)	0.2 $^a^
Agarwal et al.[26]	USA	2010	8.80	13.20 (Gabapentine)	1 $^a^
Hogl et al.[27]	Austria	2010	_	17.20 (Transdermal rotigotine)	N/A^b^
Walters et al.[28]	USA	2004	8.70	11.20 (Ropinirole)	0.5 $^a^
Aizuwa et al.[29]	Japan	2005	_	14.80 (Cabergoline)	4 $^a^

^a^ $: US dollar

^b^ N/A: Not Available

Selenium in daily recommended dose of 50 µg declines the score of RLS largely which may be due to improvement in the dopaminergic pathway (reverse thyrosine hydroxylase activity and prevention of the striatal dopamine depletion). Finally due to absence of side effects reported form selenium consumption, we can recommend selenium administration next to or instead of dopamine agonist for RLS symptoms improvement.
